# Fine mapping and identifying candidate gene of *Y* underlying yellow peel in *Cucurbita pepo*

**DOI:** 10.3389/fpls.2023.1159937

**Published:** 2023-04-21

**Authors:** Jianqing Niu, Qiong Chen, Xiaonan Lu, Xueqiang Wang, Zhongli Tang, Qinghua Liu, Fengjin Lei, Xiaoyong Xu

**Affiliations:** ^1^ Hainan Yazhou Bay Seed Lab, Sanya, Hainan, China; ^2^ College of Horticulture, Shanxi Agricultural University, Taigu, Shanxi, China; ^3^ Cotton Research Institute, Shanxi Agricultural University, Yuncheng, Shanxi, China

**Keywords:** peel color, bulked segregated analysis, *CpCHLH*, zucchini, copy number

## Abstract

As a conspicuous trait, peel color is one of the most important characteristics that affects commodity quality and consumer preferences. The locus *Y* underlying yellow peel in *Cucurbita pepo* (zucchini) was first reported in 1922; however, its molecular mechanism is still unknown. In this study, a genetic analysis revealed that yellow peel is controlled by a single dominant genetic factor. Furthermore, *Y* was mapped in a ~170 kb region on chromosome 10 by bulked segregated analysis (BSA) and fine mapping in F_2_ and BC_1_ segregating populations. The candidate region harbors fifteen annotated genes, among which *Cp4.1LG10g11560* (*CpCHLH*) is regarded as a promising candidate gene. *CpCHLH* encodes a magnesium chelatase H subunit involved in chlorophyll biosynthesis, and its mutation can result in a reduction in chlorophyll content and yellow phenotype. Interestingly, a large fragment (~15 kb) duplication containing incomplete *CpCHLH* was inserted in the candidate interval, resulting in two reformed CpCHLH proteins in the yellow parental line. It is most likely that the reformed CpCHLH proteins act as a malfunctional competitor of the normal CpCHLH protein to interrupt the formation of chlorophyll. Overall, the isolation of *Y* will shed light on the molecular mechanism of the peel color regulation of zucchini and lay a foundation for breeding.

## Introduction

*Cucurbita* (2*n* = 2*x* = 40) belongs to the family of Cucurbitaceae, which originated from the American continent and spread around the world (Paris, 1989; Bisognin, 2002; Xue et al., 2022). There are five cultivated species in *Cucurbita* (*Cucurbita pepo*, *Cucurbita maxima*, *Cucurbita moschata*, *Cucurbita argyrosperma*, *and Cucurbita ficifolia*), of which *Cucurbita pepo* (zucchini), *Cucurbita moschata*, and *Cucurbita maxima* are cultivated worldwide (Paris, 2017). *Cucurbita pepo* has the advantages of strong adaptability and a great market demand for its fruit, seed (seed oil and seed extract), and even flower, making it economically important (Xiang et al., 2018).

Peel color is one of the most conspicuous traits in zucchini, which can affect commodity quality and consumer preferences. The peel color of zucchini is highly polymorphic, and it can be mainly grouped into whites, yellows, and greens in different intensities, hues, and patterns (Sinnott and Durham, 1922; Paris, 2002). Crossing experimental results showed that white is dominant to yellow and yellow to green (Sinnott and Durham, 1922). Furthermore, peel color is a complex quantitative character, which is regulated by multiple factors. Up to now, more than a dozen loci have been identified as regulators of peel color in zucchini, including *B* (*Bicolor*), *D* (*Dark*), *Ep-1* (*Extender of pigmentation-1*), *Ep-2*, *I-mc* (*Inhibitor of mature* fruit color), *l-1* (*light fruit coloration-1*), *l-2*, *mo-1* (*mature orange-1*), *mo-2*, *pl* (*plain light* fruit color), *qi* (*quiescent intense*), *W* (*Weak* fruit coloration), and *Y* (*Yellow* fruit color) (Paris et al., 2013; Paris and Padley, 2014). Among these loci, *B*, *Ep-1*, *Ep-2*, *mo-1*, *mo-2*, and *Y* participate in the regulation of the yellow peel. Gene *B* turns the fruits from green to yellow and determines the bicolor pattern in time during development (Shifriss, 1955; Shifriss, 1981; Paris et al., 1985; Paris et al., 1986; Paris, 1988). *Ep-1* and *Ep-2* are two modifying factors of *B*. The dominant *Ep* gene can extend the area of precocious fruit pigmentation in the presence of *B* and has no evident effects conditioned by *B* ^+^/*B* ^+^ (Shifriss and Paris, 1981). The extent of precocious fruit pigmentation depends more on the dosage of these two *Ep* genes, but the dosage of *B* also plays an important role in this process (Shifriss and Paris, 1981). In addition, two *mo* genes (*mo-1* and *mo-2*) act in the loss of green fruit color prior to maturity. The plants with a combination of two recessive *mo-1* and *mo-2* can result in the loss of green fruit color, except in *L-1*/- *L-2*/- plants. The presence of either of the two dominants alone will lead to the retention of the green color (Shifriss, 1955). The main genetic factor *Y* underlying yellow peel was first reported in 1922 (Sinnott and Durham, 1922; Scarchuk, 1954; Paris et al., 2004). However, the causal gene, genetic basis, and molecular mechanism of *Y* are still poorly understood.

In addition, several genetic factors related to yellow peel color were also identified in Cucurbitaceae. For example, *CmKFB*, encoding a Kelch domain-containing F-box protein, negatively regulates the accumulation of naringenin chalcone and diverts flavonoid metabolic flux. A 12 bp insertion in the 5’ untranslated region results in hardly any expression of *CmKFB* and promotes the accumulation of yellow flavonoid pigment in muskmelon (Feder et al., 2015; Zhao et al., 2019; Ma et al., 2021). In cucumber (*Cucumis sativus*), the transcription factor MYB36 (*Csa2G352940*) was demonstrated to be the casual genetic factor of yellow-green peel (*ygp*) fruit and was predicted to interact with pigment synthesis protein (CsMYC2) to regulate the yellow-green peel coloration (Hao et al., 2018; Gebretsadik et al., 2021). Another single dominant gene *B* underlying the orange mature fruit color was mapped by QTL (quantitative trait locus) mapping in cucumber (Li et al., 2013). Further studies demonstrated that *CsMYB60* is the best candidate for *B* locus, which promotes the synthesis of flavonols and proanthocyanidins in cucumber (Liu et al., 2019; Gebretsadik et al., 2021).

Yellow-peel zucchini has higher carotenoid and lower chlorophyll content than zucchini of other colors (Paris et al., 1989; Xu et al., 2021), and it may be a potential carotenoid fortified fruit. Interestingly, Yang et al. (2022) have made significant progress in generating different colored tomato fruits by editing the genetic factors involved in the pigments synthesis (*PSY1*, *MYB12*, and *SGR1*). Although much progress has been made in the genetic analysis and characterization of the loci underlying peel color, the underlying gene and genetic mechanism are still unknown. Therefore, fine mapping and cloning of the genes regulating this trait will provide important theoretical and practical implications for zucchini breeding and custom-tailored colorful zucchini (as with tomato) in the future. In this study, we fine-mapped the major gene *Y*, regulating yellow peel in zucchini into a ~170 kb region, and developed a diagnostic InDel marker co-segregated with *Y*. Furthermore, the complex structure variations in this region were elucidated, and the novel variation of the prospective candidate gene (*Cp4.1LG10g11560*) was characterized.

## Materials and methods

### Plant materials and field trial

The two parental lines, 19pu07 and 19pu11, used in this study were high-generation inbred lines (selfed over three generations). The parental line 19pu07 (G) showed a light-green peel color, whereas 19pu11 (Y) exhibited a yellow peel color. G and Y were crossed to develop a F_1_ population. Then, F_1_ was self-crossed to derive a F_2_ population and backcrossed with G to generate a BC_1_ population. For planting the seeds of the parental lines, the F_1_, BC_1_, and F_2_ populations were sterilized, germinated, and planted in the horticultural experimental station of Shanxi Agricultural University, Taigu, Shanxi Province, during the growth period in 2020-2021. Sixty-six individual plants from a F_2_ segregating population were selected to generate a bulked yellow pool (Y_pool, 33 plants) and green pool (G_pool, 33 plants).

All the materials mentioned above were planted with 100 cm spacing between the rows and 60 cm between each individual plant. Agronomic practices regarding fertilizer and pest control were in accordance with local practices.

### Phenotype evaluation

Considering the clearly visible peel color, the phenotypes of the parental lines and populations (F_1_, F_2_, and BC_1_) were evaluated primarily by eye at the ovary stage before pollination. Furthermore, the color difference indexes containing a*, b*, L*, and CCI were used to quantify the phenotypic data using a CM‐700D colorimeter (Konica Minolta Sensing, Inc., Osaka, Japan). The indexes of L*, a*, b*, and CCI represent lightness (from 0-100 means black to white; the darkest black at L* = 0; the brightest white at L* = 100), red-green (positive value means red, and negative value means green), yellow-blue (positive value means yellow, and negative value means blue), and 1000 × a*/(L*×b*) (Camelo and Gómez, 2004).

### Pigment content determination

The method of pigment content determination was conducted as described by Xu et al. (2021). Briefly, about 1 g of peels was obtained from the parental lines and F_1_ plants at 0, 2, 6, 10, and 20 days after pollination and cut into small pieces. Then, the peels were soaked in 15 mL 96% ethanol in the dark for one day at room temperature. After centrifugation at 3000 r/min for ten minutes, the supernatants were transferred into a cuvette with 1 cm optical path. With 96% ethanol as controls, the absorption values with three biological replicates were obtained at the wavelength of 665 nm, 649 nm, and 470 nm. Finally, the content of the pigment was calculated as the following formulas (Wellburn and Lichtenthaler, 1984): C_a_ = 13.96*D665‐6.88*D649; C_b_ = 24.96*D649‐7.32*D665; C_x.c_ = (1000*D470‐2.05*C_a_‐114.8* C_b_)/245 (where C_a_, C_b_, and C_x.c_ stand for the content of chlorophyll a, b, and carotenoid, respectively; D470, D649, and D665, representing the absorbance at the wave length of 470 nm, 649 nm, and 665 nm, respectively).

### Bulked segregant analysis by re-sequencing

A young leaf tissue of the homozygous parental lines and F_2_ individual plants was sampled to extract genomic DNA by the cetyl trimethylammonium bromide (CTAB) method (Allen et al., 2006). The qualified DNA of 33 plants with yellow and green peel was mixed with an equal amount to generate the G_pool and Y_pool. Four DNA libraries containing G, Y, G_pool, and Y_pool with an insert size of 400 bp were constructed and sequenced with the Novaseq 6000 platform, yielding a total of ~41,764,246-75,849,508 150 bp paired-end raw reads (**Supplementary Table 1**). Low quality reads and adapters were discarded from the raw reads to generate clean reads using FastP (v0.20.0) (Chen et al., 2018). The clean reads were aligned to the reference sequence of zucchini (http://cucurbitgenomics.org/v2/ftp/genome/Cucurbita_pepo/) using the module of ‘mem’ in BWA (v0.7.12) with default parameters (Li and Durbin, 2009; Montero-Pau et al., 2018). Picard (v2.17.3; https://broadinstitute.github.io/picard/ ) was used to mark and remove the duplicated reads generated by PCR amplification. Variants were called using the module of ‘HaplotypeCaller’ in the Genome Analysis Toolkit (GATK, v4.0.4.0) (McKenna et al., 2010). To further evaluate the effect of the variants (including SNPs and InDels), we annotated them with the software of SnpEff using gene models in the zucchini genome assembly (De Baets et al., 2012; Montero-Pau et al., 2018). To identify the genomic region underlying *Y*, we calculated the SNP-index and ΔSNP-index for the whole genome sites. The SNP-index was defined as the ratios of reads harboring alleles to total reads at each variant site. The ΔSNP-index was derived by subtraction of the SNP-index of the Y_pool from that of the G_pool. (Abe et al., 2012; Wei et al., 2020). The values of the SNP-index and ΔSNP-index were plotted in R software.

### Molecular marker development and genotyping

As described above, the whole-genome DNA of the parental lines was re-sequenced using the Novaseq 6000 platform, and the variants were obtained by bioinformatic analysis. The insertions and deletions (InDels) between the parental lines in the candidate region were used to develop molecular markers. The polymorphism of the markers was tested in the parental lines and F_1_ plants. The PCR amplification was performed in a 10 μl system containing 1 μl DNA template (~50 ng/μl), 0.5 μl of 10 μM sense and antisense primers, 3 μl H_2_O, and 5 μl 2 × Taq PCR Starmix (GeneStar, China). The PCR was conducted as follows: 5 min at 94°C; 30 cycles of 30 s at 94°C; 30s at 52°C; 5 min at 72°C. The PCR products were separated on 12% non-denaturing PAGE gels. Only the co-dominant InDel markers were used to genotype the F_2_ and BC_1_ population. The primers used in this study are listed in **Supplementary Table 2**.

### Sequence comparison of candidate gene in parental lines

To investigate the sequence characteristics of the candidate gene in the Y parental line, we aligned the sequence of *Cp4.1LG10g11560* against the newly assembled sequences of the Y parental line based on Oxford Nanopore Technologies (ONT) in our laboratory (Contig level). The best hits were used to identify the polymorphic variants between the two parental lines. Furthermore, the polymorphic variants were validated using PCR and Sanger sequencing. Based on the sequence of *Cp4.1LG10g11560* in the reference genome, the specific primers were designed using Primer-BLAST in NCBI. The sequences of *Cp4.1LG10g11560* from the parental lines were obtained using KOD (Toyobo, Osaka, Japan) in a 25 μl volume containing 2.5 μl of 10 × PCR buffer, 2.5 μl of 2 mM dNTPs, 1.5 μl of 25 mM MgSO4, 1.5 μl each of 10 μM PCR primer, 200 ng template DNA, and 1 μl KOD DNA polymerase. The PCR amplification conditions were as follows: a hot start at 94°C for 5 min, followed by 32 cycles of 15 s at 94°C, 30 s at the primer annealing temperature and 3 min at 68°C, and final extension at 68°C for 5 min. The PCR products were sequenced by the Sanger method. A multiple sequence alignment was performed using the software of DNAMAN and SeqMan.

To test whether new transcripts were generated in the Y parental line, we blasted the sequence of *Cp4.1LG10g11560* against the full-length transcriptome data generated by PacBio technology. The best hits were used to predict the transcripts in the Y parental line.

The sequence difference of a 55 bp InDel in the second intron of *CpCHLH* between the parental lines was developed into a diagnostic molecular marker (indel1-F: TCAAATAAGCAGCCGTTC; indel1-R: AAGGCATCGTACCAATAA) to determine yellow and green peel in a natural population.

## Results

### Phenotypic analysis of peel color

**Figure 1:**
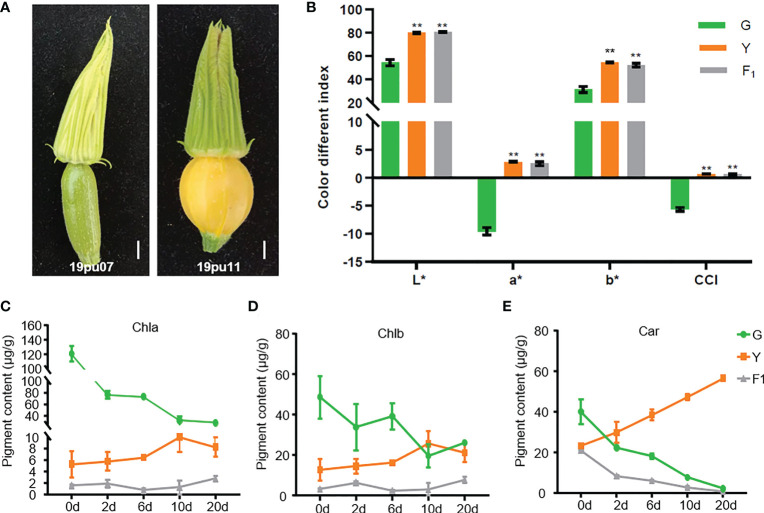
The phenotypic characteristics of the parental lines and F_1_. **(A)** The phenotype of the green (19pu07) and yellow (19pu11) parental lines. The quantified color different index **(B)**, chlorophyll a **(C)**, b **(D)**, and carotenoid content **(E)**, respectively. ** stands for significant difference at 0.01 level; and * is accompanied with L, a, and b to represent lightness, red-green, and yellow-blue, respectively.

The peel color performance of the two parental lines was investigated and quantified by a colorimeter at the ovary stage before pollination. As shown in **Figure 1A**, the peel color of parental line 19pu07 (G) exhibited light-green, while 19pu11 (Y) showed yellow. The color different indexes (represented by a*, b*, L*, and CCI) of Y were higher than that of G. Furthermore, the F_1_ generated by two parental lines showed higher color different indexes than that of G, but it was approximately equal to that of Y (**Figure 1B**). These results suggest that Y is brighter, redder, and yellower than G, and yellow peel color is dominant over green.

It is well-known that chlorophyll and carotenoid are the two primary pigments in plants, and their content ratio ultimately determines the color of the plant (Ma et al., 2021; Xu et al., 2021). To classify the underlying factors resulting in different peel colors in G and Y, we determined the content of chlorophyll (a and b) and carotenoid. The content of chlorophyll a and b in G was higher than that of Y and F_1_ at 0 days after pollination (one day before pollination), while the content of carotenoid was the opposite (**Figures 1C–E**). These results suggest that the different content of chlorophyll and carotenoid was the main reason for the different peel color at the ovary stage. Furthermore, the content of chlorophyll was decreased in G, while it slightly increased in Y and F_1_, with increasing days after pollination (**Figures 1C, D**). The content of carotenoid exhibited an entirely different trend in G and Y, with increasing days after pollination (**Figure 1E**). At the same time, the content of chlorophyll a and b in G was higher than that of Y (with the exception of Chlb on the tenth day), while the content of carotenoid was lower in G than in Y within twenty days after pollination. These results are consistent with the coloration of these accessions. Interestingly, the content of carotenoid in F_1_ also decreased with increasing days after pollination, although it showed yellow peel color. This phenomenon could be explained by the fact that F_1_ showed a faint yellow compared with Y. There may be another unknown genetic factor also involved in regulating peel color in F_1_.

### Genetic analysis and primary mapping of *Y*

The genetic linkage analysis was conducted in F_2_ and BC_1_ populations. Among 623 individual plants in the F_2_ population, 443 and 180 showed yellow and green peel color, respectively (**Table 1**). This segregation ratio fits a 3:1 ratio (χ<σπ>2</σπ> = 2.2039 < χ<σπ>2</σπ>_0.05_= 3.84; *P* = 0.138). The BC_1_ population was segregated in a 35:36 ratio for yellow peel and green peel, respectively, following a 1:1 segregation ratio (χ<σπ>2</σπ> = 0.007 < χ<σπ>2</σπ>_0.05_= 3.84; *P* = 0.933). Based on this, we speculated that the yellow peel color was controlled by a single dominant nuclear gene.

**Table 1:** Genetic analysis of peel color in parental lines and genetic populations.

Line or population	Total No. of plants	No. of yellow peel plants	No. of green peel plants	Chi-square value	*P-*value
19pu11 (Y)	30	30	0	–	–
19pu07 (G)	30	0	30	–	–
F_1_	30	30	0	–	–
F_2_	623	443	180	2.2039	0.1377
BC_1_	71	35	36	0.007	0.933

**Figure 2:**
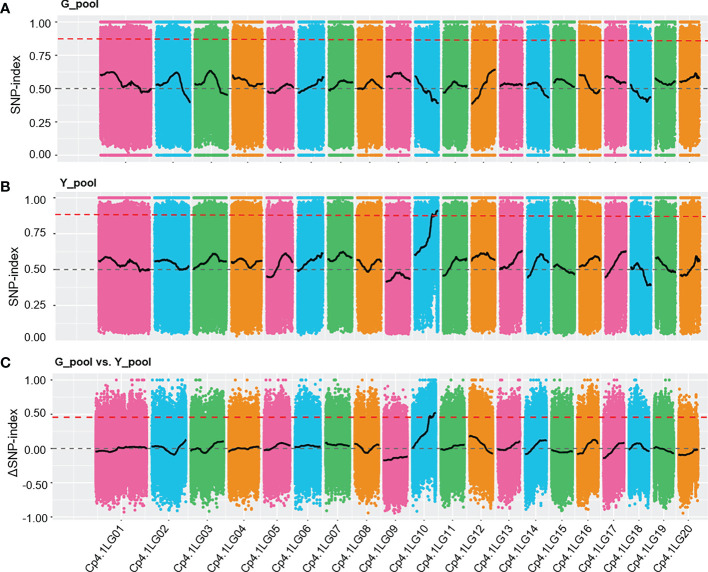
SNP-index graphs of the G_pool **(A)**, Y_pool **(B)**, and ΔSNP-index **(C)** from BSA-Seq. The red lines show the fluctuation of the average SNP-index values. The dashed grey lines represent 0.5 and 0 of the SNP-index values and ΔSNP-index value, respectively. The thresholds of 0.875 and 0.5 (red dashed lines) were used to identify significant different distribution patterns for the SNP-index and ΔSNP-index, respectively.

To rapidly identify the *Y* locus underlying yellow peel color, we used the BSA-Seq method to locate this locus. Two bulked pools of genomic DNA were obtained by mixing yellow (33) and green (33) peel plants from F_2_ generations (thereafter named Y_pool and G_pool). The two bulked pools were sequenced yielding more than an 11 Gb clean base for each pool. The ratio of the bases with a quality of Q30 or higher was more than 92.26%, which indicated the high quality of the data (**Supplementary Table 1**). More than 97.07% of the clean reads were successfully aligned to the reference, with an average coverage of 22.85× and 34.38× for the parental lines and F_2_ generation, respectively (**Supplementary Table 3**). A total of 1,796,070 and 1,807,893 SNPs were identified in the G_pool and Y_pool, respectively, which were used to calculate the SNP-index and ΔSNP-index. We detected significantly different distribution patterns (SNP-index > 0.875) of the SNP-index between the Y_pool and G_pool in the region of 7.08 Mb to the end of chromosome LG10 (**Figures 2A, B**). Furthermore, the ΔSNP-index of most the genomic regions was close to zero, whereas it was significantly higher (> 0.5) from 7.08 Mb to the end of chromosome LG10 (**Figure 2C**). This result reveals that there is a genetic factor regulating peel color in this region.

### Fine mapping of *Y* underlying yellow peel

**Figure 3:**
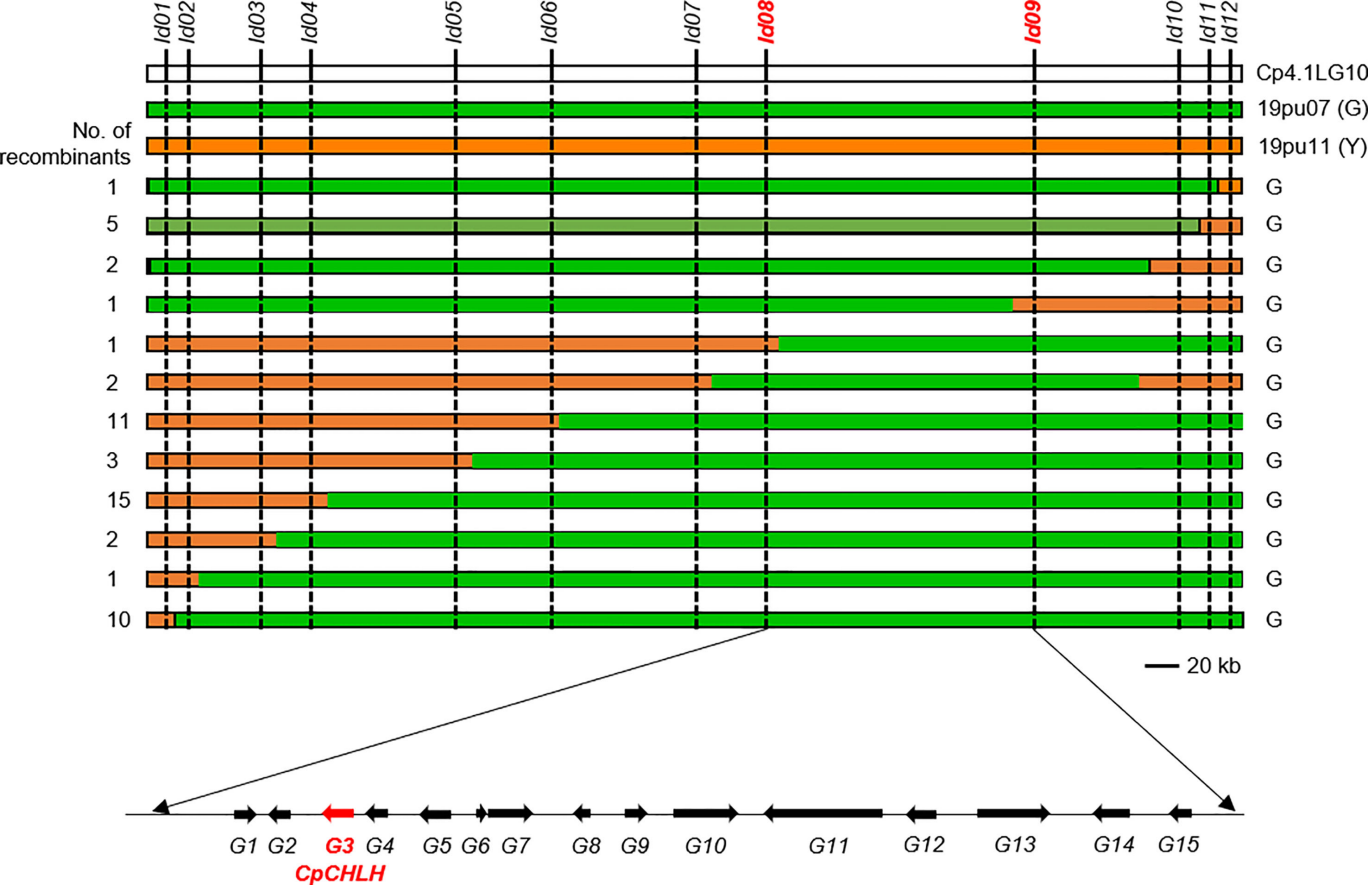
Mapping of *Y* locus. Considering *Y* is a dominant genetic factor, we mainly used the green individuals to delimit *Y*, and the phenotype is labeled on the right of the figure. The number of recombinants is labeled on the left. The bold red markers stand for the flank markers of the candidate region. The candidate gene *CpCHLH* (*Cp4.1LG10g11560*) was marked bold red.

To further confirm the results of BSA-Seq, we developed seven polymorphic InDel markers covering the candidate region to screen the F_2_ population containing 223 individuals. We delimited *Y* in a ~753 kb region flanked by markers *Id01* and *Id12* and found that the other five markers (*Id02*, *Id03*, *Id04*, *Id05*, and *Id11*) showed good linkage relationships with the peel color, suggesting the reliability of the BAS-Seq results. To further narrow down the candidate region and identify the candidate gene, we screened a bigger F_2_ and BC_1_ population containing 1,132 individual plants using the flanking markers *Id01* and *Id12*. The recombinants between *Id01* and *Id12* were genotyped using other markers and five newly developed markers (*Id6-10*). Finally, we narrowed *Y* into a ~170 kb region (8,812,267-8,983,006 bp) flanked by markers *Id8* and *Id9* (**Figure 3**).

### Candidate gene analysis

According to the genome reference sequence of zucchini (Montero-Pau et al., 2018), there were 15 annotated genes in the candidate region (*G1-G15*; **Supplementary Table 4**). *G4* and *G5* encoded the subunit of the exosome (RRP43), and *G9*, *G10*, and *G11* encoded the members of the ABC transporter A family. There were four encoded enzymes, including magnesium chelatase (*G3*), tRNA-uridine aminocarboxypropyltransferase (*G6*), Thioredoxin reductase (*G13*), and EGF domain-specific O-linked N-acetylglucosamine transferase (*G15*). There were two encoded transcription factors, including heat stress transcription factor (*G12*) and MADS-box (*G14*). Other genes encoded villin (*G7*), transmembrane protein (*G8*), and unknown proteins (*G1* and *G2*). According to the resequencing data, all 15 candidate genes harbored multiple single nucleotide polymorphic sites (SNPs) between G and Y, 13 of which harbored non-synonymous SNPs. In addition, 14 genes harbored InDels (**Supplementary Table 4**).

**Figure 4:**
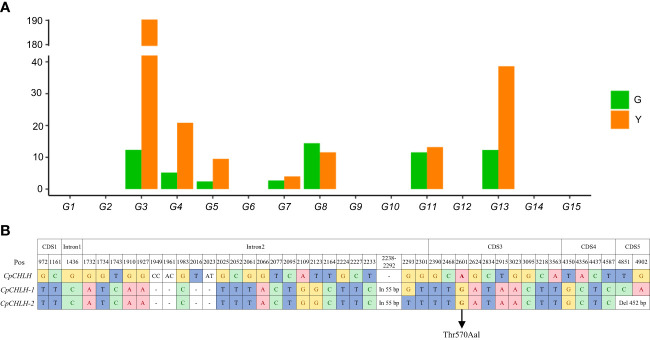
Candidate gene analysis. The expression level of candidate genes in peels **(A)** and sequence differences of *CHLH* between green and yellow parental lines **(B)**.

To further identify the putative candidate gene, we investigated the expression level of these candidate genes in the peels of the G and Y parental lines using the published RNA-Seq data (Xu et al., 2021; **Figure 4A**). The expression level of 8 genes (*G1*, *G2*, *G6*, *G9*, *G10*, *G12*, *G14*, and *G15*) was very low in both parental lines. Among the other 7 genes, 3 genes (*G7*, *G8*, and *G11*) were not significantly changed between the parental lines, while 4 genes (*G3*, *G4*, *G5*, and *G13*) were significantly up-regulated in Y. Therefore, these four genes were identified as promising candidates for determining the color of fruit peel.

### The promising candidate genes analysis

Loss of the exosome complex component led to curled leaves and much smaller rosettes in *Arabidopsis*, but it did not affect the color of the leaves, flowers, and fruits (Sikorska et al., 2017). Therefore, *G4* and *G5* may not be the target genes we are looking for. *OsCHLH*, a homolog of *G3* in rice, is involved in chlorophyll biosynthesis. The mutant of *OsCHLH*, lacking chlorophyll in the thylakoids, demonstrated chlorosis symptoms (Jung et al., 2003; Goh et al., 2004). Mutations of the *G3* homologous gene in tobacco (*Nicotiana tabacum*) and pepper (*Solanum Psedocapsicum*) resulted in significant reduction in chlorophyll and showed a yellow phenotype (Papenbrock et al., 2000; Xu et al., 2018). Considering these, we regarded *G3* as a promising candidate and renamed it as *CpCHLH* in the green peel parental line. There were abundant variations in *CpCHLH* between the two parental lines (G vs. Y), most of which were synonymous mutations or in introns (**Figure 4B**). The only non-synonymous mutation (from A to G) between G and Y was located in the third exon of *CpCHLH*, resulting in threonine to alanine (Thr570Aal). In addition, there was a 55 bp insertion in the second intron of the Y parental line (**Figure 4**). Based on this insertion, we developed an InDel marker (indel1; **Supplementary Table 2**) and found it was linked with the color of fruit peel in a small natural population containing 37 germplasms (**Supplementary Table 5**).

**Figure 5:**
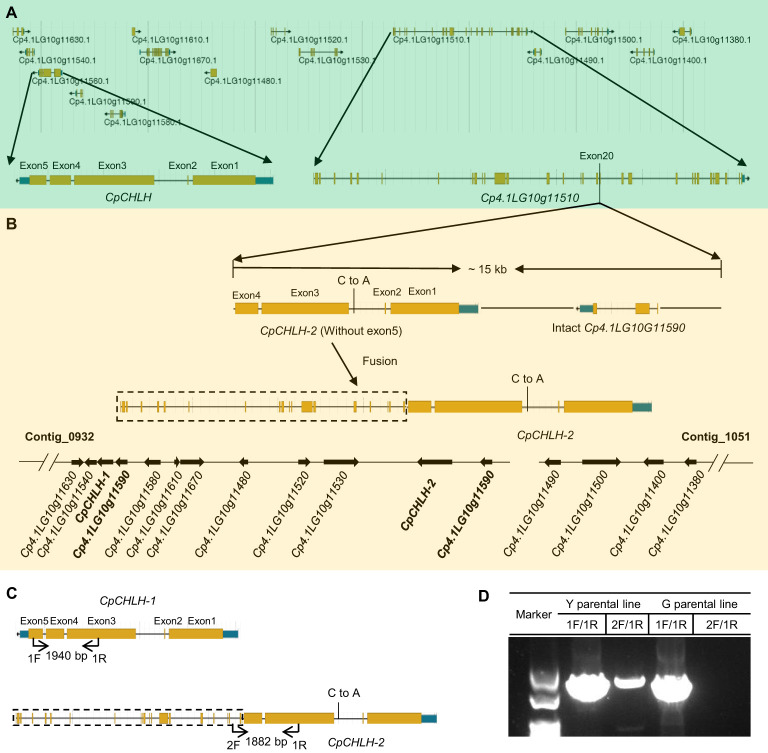
The candidate region contains complicated variations. The details of genes in the candidate region in green **(A)** and yellow **(B)** zucchini. A large fragment duplication containing incomplete *CpCHLH* (without exon5) and intact *Cp4.1LG10G11590* was inserted in *Cp4.1LG10G1150* in yellow zucchini. For simplicity, we named *Cp4.1LG10G115560* as *CpCHLH* in green zucchini and named the two copies of *Cp4.1LG10G115560* as *CpCHLH-1* and *CpCHLH-2* in yellow zucchini, respectively. There is a nucleotide substitution in the second intron between *CpCHLH-1* and *CpCHLH-2*. The specific primers were designed to verify the structure variation in yellow zucchini **(C)**, and the results were shown in agarose gel **(D)**. The primer pair of 1F/1R was designed to amplify *CpCHLH* and *CpCHLH-1* (1940 bp) in green and yellow zucchini, and 2F/1R was designed to amplify *CpCHLH-2* (1882 bp) in yellow zucchini.

**Figure 6:**
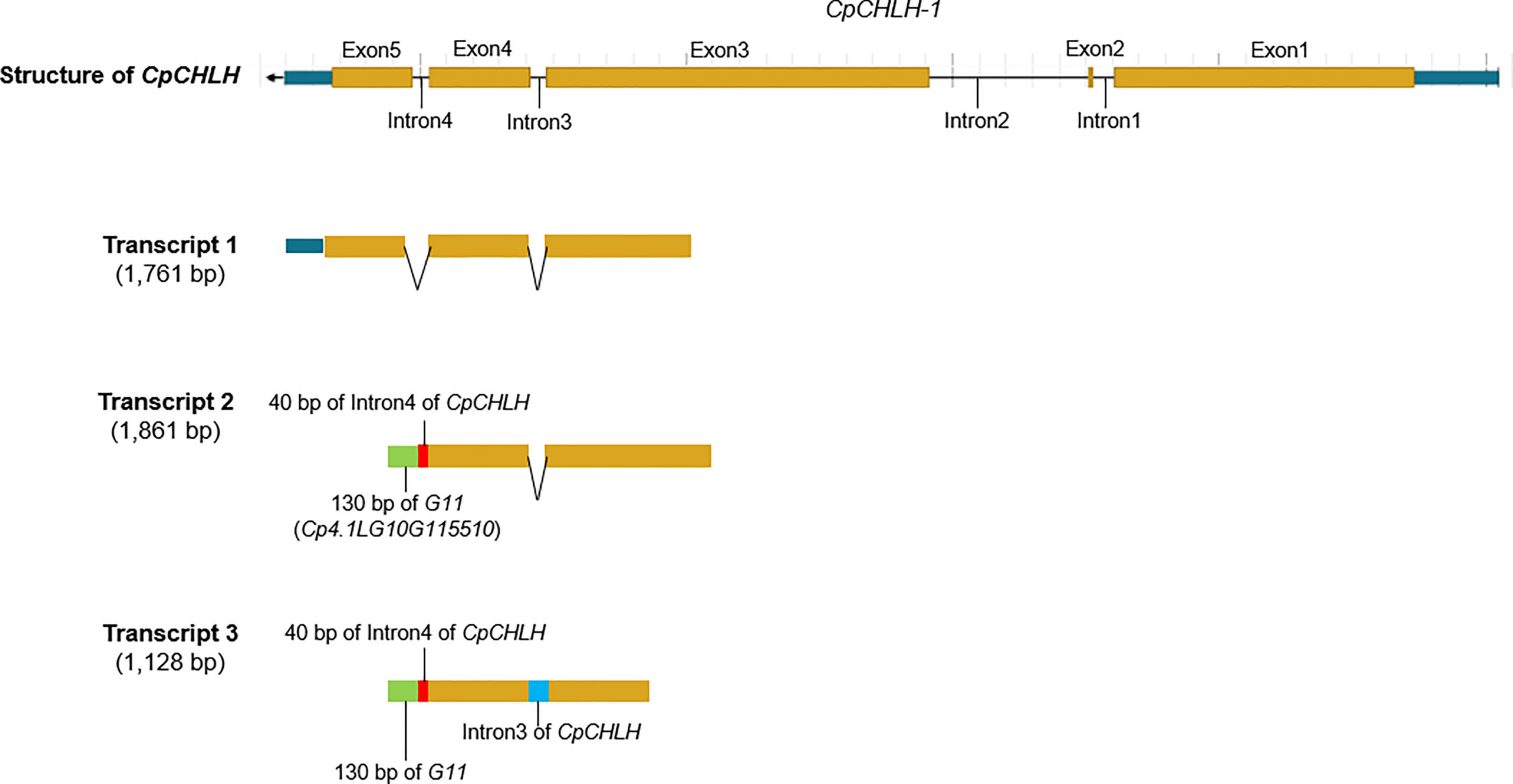
The partial-length transcript related to *CpCHLH* in the yellow parental line based on PacBio full-length transcriptome sequencing. The partial-length transcript 1 corresponds to the normal transcript annotated in the reference genome. The partial-length transcript 2 merges 40 bp of Intron4 of *CpCHLH*, 130 bp of *G11* (*Cp4.1LG10G115510*), and exons of *CpCHLH* (the first four exons). The partial-length transcript 3 merges 40 bp of Intron4 of *CpCHLH*, 130 bp of *G11*, Intron3 of *CpCHLH*, and exons of *CpCHLH* (the first four exons). The different parts of the transcripts are marked with different colors.

More interestingly, we found there was a ~15 kb duplication, containing incomplete *CpCHLH* (without the fifth exon) and intact *Cp4.1LG10g11510*, inserted into *G11* in the Y parental line based on the newly assembled sequence of yellow-peel zucchini using Oxford Nanopore Technologies (ONT) in our laboratory (Contig level; **Figures 5A, B**; **Supplementary Table S6**). Thereafter, we named the complete *CpCHLH* and incomplete *CpCHLH* as *CpCHLH-1* and *CpCHLH-2* in the Y parental line, respectively. To further confirm these results at the DNA and mRNA levels, we amplified and sequenced the full sequences of *CpCHLH-1* and *CpCHLH-2* in the two parental lines using specific primers (**Supplementary Table 2**). The results showed that *CpCHLH-1* could have been successfully obtained in the G and Y parental lines, while incomplete *CpCHLH-2* was only obtained in the Y parental line (**Figure 5C**). At the mRNA level, we investigated the full-length transcriptome based on the PacBio sequencing technology and found there existed three different transcripts, and two of them partially merged *CpCHLH* and *G11* (**Figure 6** and **Supplementary Table 6**). In addition, there was a nucleotide substitution from C to A in the second intron of *CpCHLH-2* compared with *CpCHLH-1* (**Figure 5B**). Overall, *G3*/*CpCHLH* might be the gene responsible for the yellow peel in the Y parental line.

## Discussion

Peel color is one of the major characteristics that directly influences the quality and value of fruits. There are many loci that have been reported underlying the peel color in *Cucurbita pepo*; however, none of them have been cloned. *Y* is a major locus regulating the yellow peel color, and it has been 101 years since it was first reported in 1922 (Sinnott and Durham, 1922; Scarchuk, 1954; Paris et al., 2004). However, we have little knowledge about the underlying gene and genetic mechanism of *Y*. In this study, we identified a dominant gene *Y* that regulates peel color, and it was fine-mapped onto a ~ 170 kb region by map-based cloning. According to the reported function, sequence, and expression analyses, *CpCHLH* was identified as a promising candidate gene for *Y*. However, 170 kb is a relatively large region for determining causal genes and variations; as such, a genetic transformation will be needed to confirm the gene *Y*. The cloning of this gene will shed light on the molecular mechanism of peel color regulation of zucchini and facilitate the breeding of zucchini with high nutritional value and commercial value.

### *Y* is a single dominant genetic factor underlying yellow peel color in zucchini

QTL and genes regulating fruit peel color have been identified or cloned in various crops, such as pear, apple, watermelon, melon, tomato, and cucumber (Feder et al., 2015; Yin et al., 2016; Dou et al., 2018; Han et al., 2018; Zhao et al., 2019; Gebretsadik et al., 2021; Ma et al., 2022). Among them, some QTL/genes related to yellow peel color have been characterized. For example, *CsMYB36* was proved to be involved in regulating the yellow-green peel coloration in cucumber (Hao et al., 2018). *CmKFB*, a Kelch domain-containing F-box protein-coding gene, promotes the accumulation of yellow flavonoid pigment, leading to yellow peel in muskmelon (Feder et al., 2015; Zhao et al., 2019). In these studies, the yellow peel was controlled by a single recessive nuclear gene. However, in our study the yellow peel color in zucchini was found to be regulated by a single dominant nuclear genetic factor, suggesting this might be a novel locus related to peel coloration. Interestingly, Dou et al. (2018) identified a co-located locus underlying yellow skin on chromosome 4 in watermelon by BSA-Seq and GWAS, and yellow was found to be dominant to green. Furthermore, we aligned the candidate gene *CpCHLH* against the reference genome of watermelon 97103, and we found it best matched to Cla97Chr04: 288,544-291,582, which exactly co-located with the reported results. In this region, *Cla97C04G068530.1* was also annotated as a magnesium chelatase, suggesting the casual gene for yellow peel was also *CHLH* in watermelon.

*CHLH* encodes a H subunit of magnesium chelatase that is one of the most crucial rate-limiting enzymes on the chlorophyll biosynthesis pathway (Hansson et al., 2013). It also acts as plastid-to-nucleus retrograde signaling and an abscisic acid (ABA) receptor (Mochizuki et al., 2001; Shen et al., 2006). The expression pattern of *CHLH* suggests that it is constitutively expressed in roots, stems, leaves, flowers, and fruits in tomato and *Arabidopsis* (Shen et al., 2006; Li et al., 2022). Furthermore, the mutant of *CHLH* has showed a reduction in chlorophyll content and an impaired grana lamella and thylakoid structure in chloroplast, leading to a yellow leaf phenotype in barely, tobacco, etc. (Jensen et al., 1996; Papenbrock et al., 2000; Xu et al., 2018). However, in this study, the Y parental lines showed yellow peel and flesh, while the leaves, stems, and other organisms were of green color. This phenomenon was also reported in tomato, in which the *SlCHLH* mutant showed yellow stigma but green leaves and other tissues (Li et al., 2022).

### The possible genetic mechanism underlying *Y*

In the candidate region, we found a large fragment duplication (~15 kb) containing incomplete *CpCHLH* and intact *G4* inserted in *G11*, which leads to the function disruption of *G11* and the fusion of *G11* (the first nineteen exons) and *CpCHLH-2* (**Figure 5**). Although the large fragment duplication leads to the disruption of *G11* and an increase in the copy number of *G4*, they showed little probability to be the causal gene for yellow peel, based on the expression level, functional annotation, and roles of their homologs in *Arabidopsis*. Meanwhile, *CpCHLH* encodes a subunit H of Mg-chelatase, and mutations in *CHLH* lead to an underdeveloped thylakoid member, low chlorophyll content, and a chlorosis phenotype in rice and other organisms (Goh et al., 2004; Li et al., 2022; Zhang et al., 2022). Interestingly, there are three transcripts related to *CpCHLH* in Y parental line, and two of them could be translated to gain-of-functional proteins. Based on this, we thought the reformed *CpCHLH* is most likely the causal gene. Furthermore, we proposed the possible genetic mechanism underlying reformed *CpCHLH* for yellow peel. First, the reformed CpCHLH might serve as a malfunctional competitor with normal CpCHLH interrupting chlorophyll formation. Then, the malfunctional Mg-chelatase protein complex results in chlorophyll reduction and yellow peel color. Second, apart from the catalyzing function, CHLH also participates in the plastid–nuclear signaling pathway that regulates the expression of photosynthesis-related nuclear genes in *Arabidopsis* (Mochizuki et al., 2001; PNAS). Based on this, another possible mechanism underlying yellow peel is that dysfunctional CpCHLH cannot regulate the downstream photosynthesis-related nuclear genes. Further experimental evidence is needed to confirm these hypotheses.

## Data availability statement

The original contributions presented in the study are included in the article/**Supplementary Material.** Further inquiries can be directed to the corresponding authors.

## Author contributions

XX conceived and supervised the project. FL provided the materials and designed the project. JN analyzed the data, plotted figures, and drafted the manuscript. XL created mapping populations, investigated the phenotypic data, and conducted BSA analysis. QC cloned the sequences of candidate gene and assisted in designing figures and drafting the manuscript. ZT provided help with fine mapping. QL provided assistance in creating materials. XW assembled the genome of the Y parental line, and XX analyzed the transcriptome data. JN, QC, and XX revised the manuscript. All authors contributed to the study and approved the final version for submission.
